# A nod to the bond between NOD2 and mycobacteria

**DOI:** 10.1371/journal.ppat.1011389

**Published:** 2023-06-01

**Authors:** Jean-Yves Dubé, Marcel A. Behr

**Affiliations:** 1 Department of Microbiology and Immunology, McGill University, Montréal, Canada; 2 Department of Medicine, McGill University Health Centre, Montréal, Canada; University of Washington, UNITED STATES

## Abstract

Mycobacteria are responsible for several human and animal diseases. NOD2 is a pattern recognition receptor that has an important role in mycobacterial recognition. However, the mechanisms by which mutations in NOD2 alter the course of mycobacterial infection remain unclear. Herein, we aimed to review the totality of studies directly addressing the relationship between NOD2 and mycobacteria as a foundation for moving the field forward. NOD2 was linked to mycobacterial infection at 3 levels: (1) genetic, through association with mycobacterial diseases of humans; (2) chemical, through the distinct NOD2 ligand in the mycobacterial cell wall; and (3) immunologic, through heightened NOD2 signaling caused by the unique modification of the NOD2 ligand. The immune response to mycobacteria is shaped by NOD2 signaling, responsible for NF-κB and MAPK activation, and the production of various immune effectors like cytokines and nitric oxide, with some evidence linking this to bacteriologic control. Absence of NOD2 during mycobacterial infection of mice can be detrimental, but the mechanism remains unknown. Conversely, the success of immunization with mycobacteria has been linked to NOD2 signaling and NOD2 has been targeted as an avenue of immunotherapy for diseases even beyond mycobacteria. The mycobacteria–NOD2 interaction remains an important area of study, which may shed light on immune mechanisms in disease.

## Introduction

*Mycobacterium* is a genus of bacteria responsible for epidemiologically important human and veterinary diseases. Tuberculosis (TB) is estimated to have killed over 1 billion people since 1882 when Robert Koch discovered the etiological agent, *Mycobacterium tuberculosis* (*Mtb*) [1,2], and TB continues killing over 1 million annually [3]. *M*. *leprae* and nontuberculous mycobacteria (NTM) like *M*. *avium* and *M*. *abscessus* cause suffering to hundreds of thousands of people each year [4]. Additionally, in the veterinary world, bovine TB (due to *M*. *bovis* and *M*. *orygis*) and bovine paratuberculosis (due to *M*. *avium paratuberculosis*, herein abbreviated as *Map*) present as health and economic challenges, both directly to livestock and due to their risk of zoonotic spread to humans [5–7]. Reliable vaccines are difficult to design for mycobacteria because the correlates of protection are unknown. To make better interventions, we need to understand the immune response to mycobacteria.

The immune response begins with the recognition of microbe-associated molecular patterns (MAMPs) by host pattern recognition receptors (PRRs). The overall importance of PRRs in mycobacterial infection seems to be limited by the redundancy of many PRRs, yet owing to their distinct signaling mechanisms, individual PRRs may alter the outcome of infection in specific, nonredundant ways with subtle, if not significant, consequences [8]. Two decades ago, a newly described gene called Nucleotide-binding Oligomerization Domain-containing 2 (*NOD2*, formerly known as Caspase Recruitment Domain 15 or *CARD15*), initially associated with susceptibility to Crohn’s disease [9–11] and Blau syndrome [12], was shown to mediate recognition of bacterial peptidoglycan (PGN) [13,14]. Not long thereafter, genetic, chemical, and immunological evidence specifically linking *NOD2* to mycobacteria mounted. Despite this, there lacks a consensus on the mechanisms by which NOD2 affects the outcome of mycobacterial infections.

To provide a foundation for moving the topic forward, herein we have combined our knowledge of NOD2 and mycobacteria with a review of the literature directly examining these elements together. We have collected 60 papers experimentally testing a relationship between NOD2 and mycobacteria (Table 1). Reflecting the topics covered in these papers, we discuss NOD2 in the context of mycobacterial infection, including expression, elicited effector functions, and outcomes in animal models, followed by a nod to the potential interventions founded on the NOD2–mycobacteria interaction. While much work remains to understand the mechanisms behind the association between NOD2 and mycobacteria, the evidence supports that this relationship has important implications for the outcome of mycobacterial infection.

**Table 1:** Research articles with data directly addressing NOD2 and mycobacteria^a^.

Reference	Year	Human model(s)	Mouse model(s)	Pathogen/NOD2 ligand(s)	NOD2 system	Readouts	^b^
**[15]** Ferwerda	2005	HEK, PBMC	PM	*Mtb* sonicate	*NOD2* reporter, *Nod2*-KO, *NOD2*fs	NF-κB, cytokine	Y
**[16]** Loeuillet	2006	THP-1	-	*Mtb*	*RIPK2* dominant negative	Apoptosis	N
**[17]** Ferwerda	2007	HEK, PBMC	-	*Map* sonicate	*NOD2* reporter, *NOD2*fs	NF-κB, cytokine	Y
**[18]** Gandotra	2007	-	BMDM, BMDC	*Mtb*	*Nod2*-KO	NO, CFU, cytokine	Y
**[19]** Lala	2007	BAL cells, PBMC +/− TB	-	Natural *Mtb* infection	Natural *NOD2*	*NOD2* expression	Y
**[20]** Srivastava	2007	-	AM	BCG	Natural *NOD2*	*Nod2* expression	Y
**[21]** Stanley	2007	-	BMDM	*Mtb*	*Ripk2*-KO	Cytokine	N
**[22]** Divangahi	2008	-	In vivo, AM	BCG, *Mtb*	*Nod2*-KO	CFU, survival, various immuno.	Y
**[23]** Leber	2008	-	BMDM	*Mtb*	*Nod2*-KO	Cytokine	Y
**[24]** Magalhaes	2008	-	In vivo	CFA	*Nod2*-KO	Cytokine, IgG	Y
**[25]** Coulombe	2009	HEK	In vivo, PM, RAW	*M*. *smeg* (Δ*namH*), MDPs (A+G)	*Nod2*-KO	Cytokine, signaling	Y
**[26]** Kleinnijenhuis	2009	PBMC	-	*Mtb*	*NOD2* fs	Cytokine	Y
**[27]** Pandey	2009	-	BMDM	*Mtb*, MDPs (A+G)	*Nod2*-KO, *Ripk2*-KO	Cytokine, signaling	Y
**[28]** Sinsimer	2010	Monocytes	-	*M*. *leprae*, *N*-acetyl MDP	Natural *NOD2*	Cytokine	Y
**[29]** van de Veerdonk	2010	PBMC	-	Heat-killed *Mtb*	*NOD2* fs	Cytokine	N
**[30]** Brooks	2011	MDM, AM	BMDM	*Mtb*, BCG, *N*-acetyl MDP	*NOD2*-KD (siRNA), *Nod2*-KO	Cytokine, signaling, CFU	Y
**[31]** Glubb	2011	Monocytes	-	*Map*	*NOD2* heterozygotes	CFU, cytokine	Y
**[32]** Kang	2011	HEK	-	*M*. *leprae*	*NOD2* overexpression	Signaling, cytokine	Y
**[33]** Shaw	2011	-	In vivo	CFA	*Nod2-KO*, *Ripk2-KO*	EAE, various immuno.	Y
**[34]** Sun	2011	Leprous skin	-	Natural *M*. *leprae* infection	Natural *NOD2*	*NOD2*, *RIPK2* expression	Y
**[35]** Abdalla	2012	-	BMDC	*Mtb*	*Nod2*-KO	Cytokine	N
**[36]** Dorhoi	2012	-	BMDM	*Mtb*	*Nod2*-KO	Cytokine	N
**[37]** Juárez	2012	AM	-	*Mtb*, *N*-acetyl MDP	Natural *NOD2*	CFU, cytokine, autophagy	Y
**[38]** Kleinnijenhuis	2012	Monocytes	-	BCG, *N*-acetyl MDP	*NOD2* fs, RIPK2 inhibitor	Training (cytokine)	Y
**[39]** Manzanillo	2012	-	BMDM	*Mtb*	*Nod1/Nod2* double KO	Cytokine, signaling	N
**[40]** Pandey	2012	-	PM	*Mip*	*Nod2-*KD	Cytokine, *Nod2* expression	Y
**[41]** Schenk	2012	Monocytes, leprous skin	-	Natural *M*. *leprae* infection, *N*-acetyl MDP	Natural NOD2	Cytokine, CD1B	Y
**[42]** Nabatov	2013	In vitro	-	Sulpholipid-1, sulfatide	Many *NOD2* deletions	Binding assay	Y
**[43]** Shenderov	2013	-	In vivo	CFA, *Mtb* PGN	*Nod1/Nod2* double KO	Cytokine	Y
**[44]** Verway	2013	THP-1	-	*Mtb*	*NOD2*-KD	Cytokine	N
**[45]** Xie	2013	Goldfish macrophages^c^		*M*. *marinum*	Natural *NOD2*	*Nod2* expression	Y
**[46]** Hansen	2014	MDM	In vivo, PM	*Mtb* (Δ*namH*), *N*-glycolyl MDP	*Nod2*-KO	Cytokine, CFU, survival	Y
**[47]** Arts	2015	Monocytes	-	γ-irradiated BCG	*NOD2* fs	Training (cytokine)	Y
**[48]** Carvalho	2015	-	In vivo, BMDM	*M*. *avium*	*Nod2*-KO	Cytokine, CFU, histology	Y
**[49]** Coussens	2015	MDM	-	*Mtb*	Natural *NOD2*	*NOD2* expression	Y
**[50]** Katsunuma	2015	-	RAW	Z-100 *Mtb* extract	*Nod2*-KD	Cytokine, *Nod2* expression	Y
**[51]** Kim	2015	HEK	Footpad, RAW	*M*. *leprae*	*NOD2* overexpression	*Nod2*, *CASP1* expression	Y
**[52]** Kim	2015	-	In vivo, BMDC	MDPs (A+G)	*Nod2*-KO	Cytokine, signaling	Y
**[53]** Landes	2015	MDM	-	*Mtb*, MDPs (A+G)	*NOD2*-KD	iNOS/NO	Y
**[54]** Salem	2015	PBMC	-	*M*. *smeg (ΔnamH)*, *Map*, MDPs (A+G)	*NOD2* variants	Cytokine, signaling	Y
**[55]** Khan	2016	-	BMDC	*Mtb*, *N*-glycolyl MDP	Natural NOD2	CFU, various immuno.	Y
**[56]** Khan	2016	-	In vivo, BMDC	*Mtb*, *N*-glycolyl MDP	Natural NOD2	CFU, various immuno.	Y
**[57]** Khan	2016	-	BMDC	*Mtb*, *N*-glycolyl MDP	Natural NOD2	Various immuno.	Y
**[58]** Lachmandas	2016	PBMC	BMDM	*Mtb*	*NOD2*fs, *Nod2*-KO	Cytokine, lactate	Y
**[59]** Lee	2016	-	BMDM	*Mtb*	*Nod2*-KO	Cytokine	Y
**[60]** Schenk	2016	Monocytes, HEK	-	*M*. *leprae* + PGN	*NOD2*-KD, *NOD2* overexpression	Cytokine, CD1b, signaling	Y
**[61]** Andreu	2017	-	BMDM, J774A.1	*Mtb*	Natural *NOD2*	*Nod2* expression	Y
**[62]** Lee	2017	-	In vivo, BMDM	*M*. *abscessus*, *N*-glycolyl MDP	*Nod2-*KO	CFU, various immuno.	Y
**[63]** Bickett	2020	-	In vivo	BCG, *Mtb*	*Nod1/Nod2* double KO	CFU	N
**[64]** Dubé	2020	-	In vivo, BMDC	CFA (Δ*namH*), MDPs (A+G)	*Nod2*-KO	Various immuno.	Y
**[65]** Khan	2020	HEK	In vivo, BMDM/DC	BCG, *Mtb*, Inarigavir	*Nod2*-KO, *NOD2* overexpression	CFU, various immuno.	Y
**[66]** Lu	2020	-	BMDM	*M*. *smeg* (Rv1096), *Mtb* (Δ*Rv1096*)	*Nod2*-KO	CFU, cytokine	Y
**[67]** Napier	2020	-	In vivo	CFA	*Nod2-KO*, *Ripk2-KO*	Uveitis	Y
**[68]** Ahn	2021	-	In vivo, BMDM	*M*. *abscessus*	*Nod2*-KO	CFU, various immuno.	Y
**[69]** Aqdas	2021	-	In vivo, BMDC	*Mtb*, *N*-glycolyl MDP	Natural *NOD2*	CFU, various immuno.	Y
**[70]** Aqdas	2021	-	Mesenchymal stem cells	*Mtb*, *N*-glycolyl MDP	Natural *NOD2*	Cytokine, CFU, autophagy	Y
**[71]** Correa-Macedo	2021	AM +/− PrEP	-	*Mtb*	Natural *NOD2*	NOD2 path activity	Y
**[72]** Mandala	2021	PBMC +/− TB	-	*N*-acetyl MDP	*NOD2* variants	*NOD2* expression	Y
**[73]** Dubé	2022	-	In vivo	*Mtb*	*Nod2*-KO, *Mincle-Nod2* double KO	CFU, survival, various immuno.	Y
**[74]** Dallmann-Sauer	2023	MDM	RAW	BCG, *N*-glycolyl MDP	*NOD2* variants, *NOD2* overexpression	ROS, apoptosis, NF-κB, cytokine	Y

^a^AM, alveolar macrophages; BMDC, bone marrow–derived dendritic cells; BMDM, bone marrow–derived macrophages; CFU, colony-forming unit (bacterial burden); HEK, HEK293(T) cells; KD, siRNA-mediated knockdown; KO, knockout; *M*. *smeg*, *M*. *smegmatis*; MDM, monocyte-derived macrophages; MDPs (A+G), *N*-acetyl and *N*-glycolyl MDP; *NOD2* fs, *NOD2* frameshift mutation; PBMC, peripheral blood mononuclear cell; PM, peritoneal macrophages; PrEP, preexposure prophylaxis; various immuno., various diverse immunological readouts tested.

^b^Contains data supporting a role for NOD2 during mycobacterial infection (Yes/No).

^c^Neither human nor mouse, but goldfish.

## How are mycobacteria and NOD2 linked?

**Fig 1:**
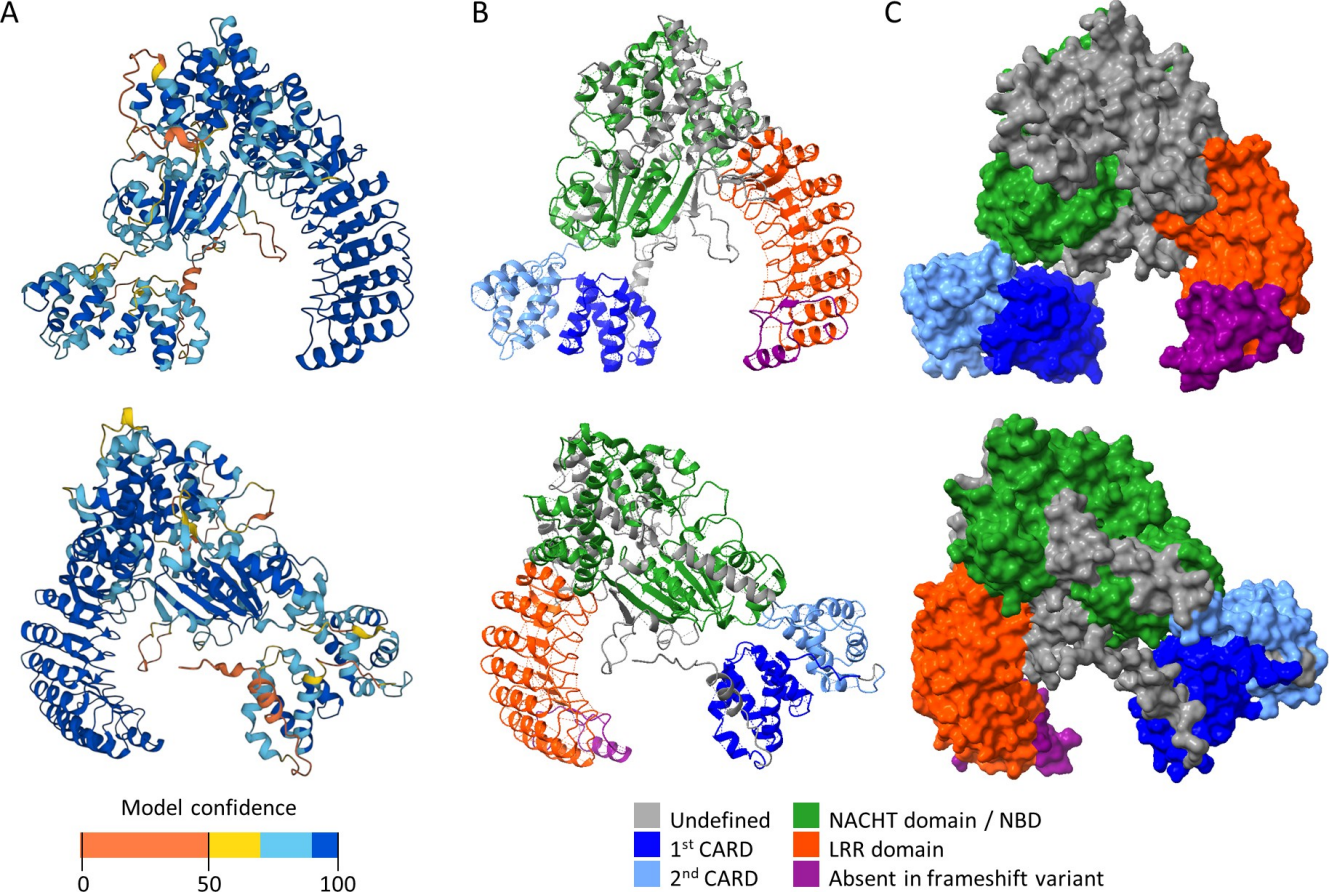
Structure of human NOD2. These structural representations were generated from UniProt entry Q9HC29 (“NOD2_HUMAN”) folded with AlphaFold. Approximately 180° of rotation separates the images from the top and bottom rows. (**A**) Ribbon structure of NOD2 with AlphaFold per-residue confidence score (pLDDT) in range from 0 to 100 (low to high confidence). (**B**) Ribbon and (**C**) space-filling models of AlphaFold NOD2 illustrated using UCSF ChimeraX based on UniProt domain annotation. NOD2 has 4 domains: 2 CARDs, 1 NACHT/NBD, and 1 LRR domain. In Crohn’s disease, some patients have a frameshift variant where the protein is foreshortened by 34 amino acids. This missing region is shown in mauve. CARD, caspase recruitment domain; LRR, leucine-rich repeat; NBD, nucleotide-binding domain; NOD2, Nucleotide-binding Oligomerization Domain-containing 2.

In 2003, NOD2 was demonstrated to be essential for the innate immune response to the common bacterial PGN motif muramyl dipeptide (MDP) [13,14]. NOD2 has 4 domains: The 2 caspase recruitment domains (CARDs) mediate interactions with other proteins; the nucleotide-binding domain (NBD, or NACHT) binds ATP, which was proposed to provide a conformational change that enhances the MDP interaction and oligomerization, while ATP hydrolysis to ADP has the opposite effect [75]; the leucine-rich repeats (LRRs) are thought to compose the ligand (MDP) binding domain [76,77]. A representation of human NOD2 is illustrated in Fig 1 [78,79]. During bacterial infection, PGN is digested in the phagosome by lysozyme to release muropeptides like MDPs [80], which traverse SLC15A transporters [81–83] to enter the cytosol where NOD2 resides. NOD2 must also localize to cellular or phagosomal membranes via palmitoylation by ZDHHC5 for MDP recognition [84–86]. Recently, the enzyme NAGK was identified to be essential for the response to MDP upstream of NOD2: NAGK phosphorylates MDP as it enters the cytoplasm, and only phospho-MDP can signal through NOD2 [87]. NOD2 binding to phospho-MDP has yet to be demonstrated. Earlier, surface plasmon resonance was used to show binding of unphosphorylated MDP to NOD2 [76,88], and tagged NOD2 was detected from a pull-down with biotin-MDP [75]. It remains to be clarified whether NOD2 binds to the phosphorylated and/or nonphosphorylated form of MDP. We refer the reader to [89,90] for more on NOD2 signaling.

### 1. The genetic link

Mutations in *NOD2* have been strongly associated with Crohn’s disease, especially the common LRR domain mutations 2104CT (R702W), 2722GC (G908R), and 3020insC (1007FS) [9–11,91,92]. These are recessive loss-of-function mutations (i.e., loss of MDP responsiveness) [13,14]. The 1001GA (R334Q) mutation in the NBD of NOD2 was associated with Blau syndrome [12]. Both Crohn’s disease and Blau syndrome are idiopathic, but the existence of a bacterial contributing agent is indirectly supported, given that NOD2 is a PGN sensor. Because of parallels to the intestinal pathology of Johne’s disease (caused by *Map* infection in ruminants), *Map* has and continues to be hypothesized to play a role in Crohn’s disease [93]. *Map* infection was reported in patients with NOD2 polymorphisms and/or Crohn’s disease [94,95], but a causative role for *Map* in Crohn’s disease is elusive. Other functions for NOD2 have been reported outside of being solely a PGN sensor, such as a role in viral RNA sensing [96,97] and the endoplasmic reticulum stress response [98–101], which do not contradict but provide alternative hypotheses to a bacterial etiology for Crohn’s disease and Blau syndrome and, therefore, must also be considered.

NOD2 interacts with the adaptor protein kinase RIPK2 for signaling [102–104], and mutations in both *NOD2* and *RIPK2* have been associated with leprosy and/or its endophenotypes [105–109]. Additionally, NOD2 was recently shown to interact with LRRK2, a kinase genetically linked with Parkinson’s disease [74]. Reports have linked NOD2 to LRRK2-dependent intestinal homeostasis and RIPK2 phosphorylation [110–112], and *LRRK2* polymorphisms are also associated with Crohn’s disease and leprosy susceptibility [74,105,113–117], indicating a common genetic network at play. Polymorphisms in the autophagy gene *ATG16L1* are also linked to Crohn’s disease like *NOD2* [118,119], suggesting another shared pathway, and the possibility of a contributing bacterial agent vis-à-vis autophagic control of the intruder.

In one study, the P268S and R702W mutations were linked with a decreased risk of TB, while the A725G mutation was associated with increased risk; all three are predicted to have an impact on the structure of NOD2, but functional data were not provided in this study [120]. Overexpressed NOD2 with R702W recently confirmed decreased signaling capacity [74]. In another study, intronic SNPs in *NOD2* (rs6500328 and rs2111234) were associated with an *Mtb* resister phenotype (persistently negative for disease and immune conversion) but not TB per se [121,122]. Recently, NOD2 was associated with TB meningitis via SKAT-O analysis [123]. Intronic/synonymous SNPs were not evaluated for expression changes in *NOD2* in these studies, which remains a possibility. Overall, the mechanism of the association between *NOD2* and TB is unclear. Evaluation of the genetic link between *NOD2* and TB may require a better definition of TB endophenotypes due to the nature of PRRs’ subtle modifications of immune responses, as has been done for leprosy [8].

*NOD2* variants have been associated in one paper with Buruli ulcer in humans (due to *M*. *ulcerans*) [124] and in another to *Map* infection in cattle [125]. Case reports have been published identifying *NOD2* mutations in patients with *M*. *abscessus* or MAC infection [126], but controlled genetic studies associating *NOD2* with these infections have not been performed.

### 2. The chemical link

**Fig 2:**
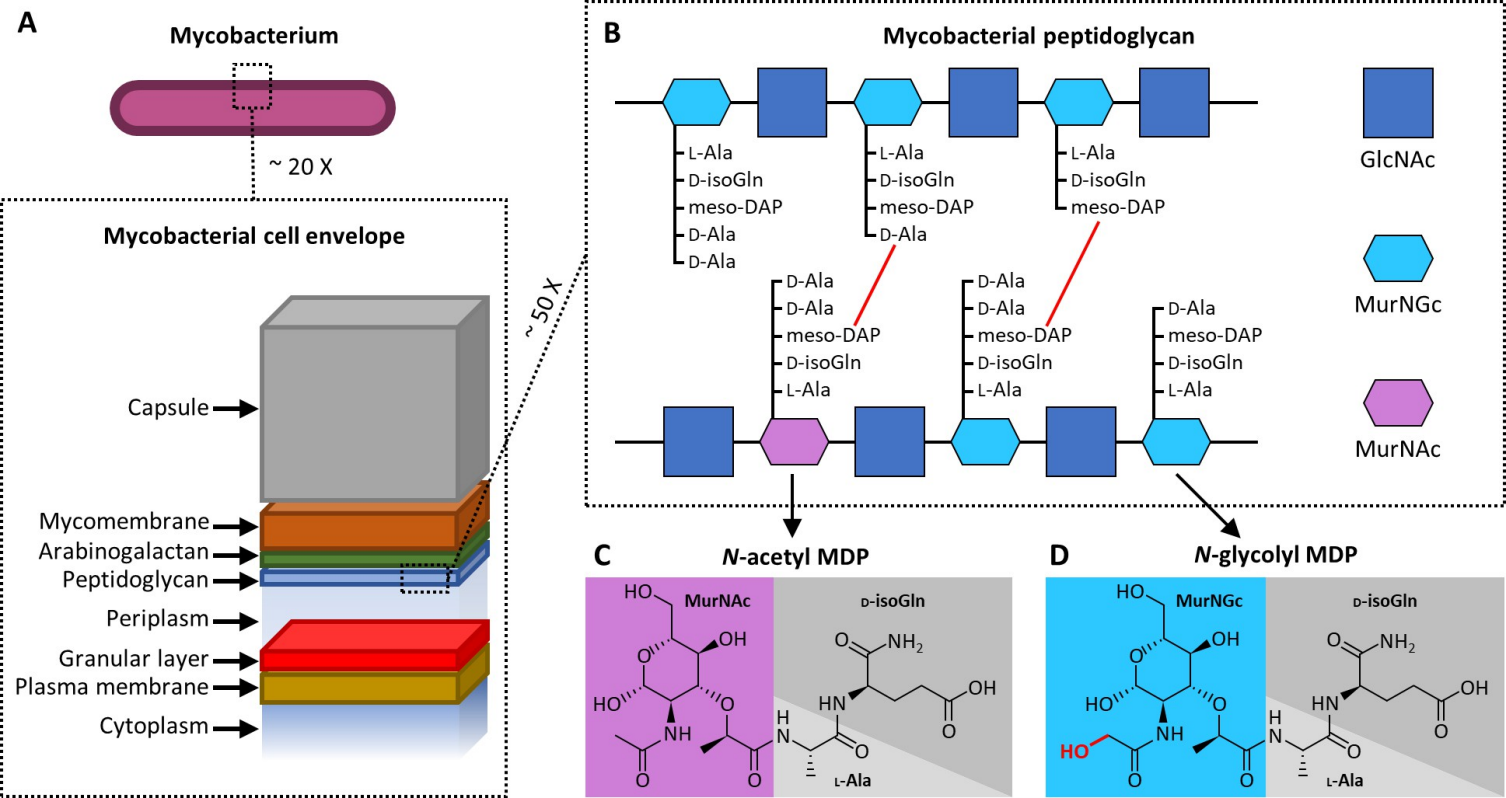
The cell envelope of mycobacteria and mycobacterial peptidoglycan. (**A**) Cross-sectional cartoon of the mycobacterial envelope, showing major regions in approximate relative size to each other. (**B**) Parallel strands of mycobacterial PGN containing MurNGc, MurNAc, and GlcNAc, with peptides partially cross-linked (red lines). (**C**) *N*-acetyl MDP, the minimal PGN fragment recognized by NOD2, common to most bacteria. The sugar and amino acids are separated by differently colored backgrounds. (**D**) *N*-glycolyl MDP, the distinct NOD2 ligand of mycobacteria, with the additional hydroxy group from *N*-glycolylation highlighted in red. The sugar and amino acids are separated by differently colored backgrounds. GlcNAc, *N*-acetyl glucosamine; MDP, muramyl dipeptide; MurNAc, *N*-acetyl muramic acid; MurNGc, *N*-glycolyl muramic acid; NOD2, Nucleotide-binding Oligomerization Domain-containing 2; PGN, peptidoglycan.

The mycobacterial cell envelope is a defining feature of the genus. Mycobacteria have an inner plasma membrane plus an outer “mycomembrane” (Fig 2A). Between these membranes are a poorly defined granular layer, the periplasmic space, PGN, and arabinogalactan [127,128]. The outermost surface of the cell is a carbohydrate capsule, which varies in composition across the genus [129,130]. Distinctly, mycobacterial PGN contains alternating units of *N*-glycolyl muramic acid (MurNGc) (or less frequently, *N*-acetyl muramic acid (MurNAc)) and *N*-acetyl glucosamine (GlcNAc) (Fig 2B). Other bacteria possess only MurNAc in the place of MurNGc. The muramic acid moiety is attached to peptides containing L-alanine (L-Ala), D-isoglutamine (D-isoGln), *meso*-diaminopimelic acid (*meso*-DAP), and occasionally 1 or 2 residues of D-alanine (D-Ala). Peptides cross-link glycan strands through DAP-DAP bonds or D-Ala-DAP bonds [131]. Muramyl *dipeptide* is the fragment containing muramic acid, L-Ala, and D-isoGln, and while most bacteria have *N*-acetyl MDP (Fig 2C), *N*-glycolyl MDP is the distinct NOD2 ligand of mycobacteria (Fig 2D). During PGN synthesis, uridine diphosphate-linked *N*-acetyl muramic acid (UDP-MurNAc) is a cytoplasmic intermediate. In mycobacteria, some of this intermediate becomes *N*-glycolylated by the enzyme *N*-acetyl muramic acid hydroxylase (NamH) using molecular oxygen to form UDP-MurNGc [132–134]. The actinobacteria genera *Nocardia* and *Rhodococcus* have *namH*, while it is absent in the *Streptococcus* and *Corynebacterium* genera [25,127,133,135]. Mycobacterial cell wall synthesis is reviewed in [131].

The presence of NamH in many nonpathogenic actinobacteria suggests that *N*-glycolylated PGN evolved in a host-free environment; supporting this, the presence of *namH* has been shown to confer resistance to beta-lactams and lysozyme [127,133]. *N*-glycolylation of PGN is nonessential for survival of mycobacteria, since it can be deleted from *M*. *smegmatis* and *Mtb* [25,46], and is degenerate in the *M*. *leprae* genome [134]. It is noteworthy that the PGN modification is retained in *Mtb*, despite thousands of years of *Mtb*–human interaction [136], yet lost in *M*. *leprae*. This divergent evolution provides indirect support that the NOD2 ligand plays important roles in the life cycles of pathogenic mycobacteria. The requirement for molecular oxygen for NamH-mediated *N*-glycolylation [132,133] is interesting, as *Mtb* experiences different concentrations of O_2_ in different zones of pulmonary lesions [137]. Indeed, *Mtb* cultured in vitro under hypoxic conditions up-regulates *namH* (*Rv3818*) expression upon returning to an aerobic environment [138]. The regulation and timing of *namH* expression may inform about the role of NOD2 sensing during *Mtb* infection and should be further studied.

The sensing of PGN can also occur via other PRRs. NOD1 senses D-glutamyl-meso-diaminopimelic acid, but its role is limited in mycobacterial infection relative to NOD2 [59]. NLRP3 inflammasome activation was shown to proceed through hexokinase sensing of *N*-acetyl glucosamine released from PGN [139], although this has not been verified in the context of mycobacterial infection. NLRP3 sensing of *Mtb* involves membrane damage and contributes significantly to the cellular immune response [140,141], but NLRP3 is not critical during *Mtb* infection of mice [142]. NLRP1 was attributed to MDP sensing [143,144] and PGN recognition proteins (PGLYRPs) contribute to detection of PGN [145–148], but none are well examined in mycobacterial infection. While we must be mindful of other PPRs when discussing the immunology of mycobacterial PGN, only NOD2 sensing is known to be specifically affected by the *N*-glycolylation of PGN in mycobacteria, and the existence of these other PRR sensors does not per se detract from the importance of NOD2.

### 3. The immunologic link

Soon after NOD2 was described as a PGN sensor, NOD2 was demonstrated to contribute to the immune response to mycobacteria. Overexpression of NOD2 in HEK293T cells was sufficient to induce NF-κB signaling upon *Mtb* and *Map* infection, and various cytokine responses to *Mtb* and *Map* sonicates were reduced in human PBMCs homozygous for the NOD2 frameshift mutation [15,17]. *NOD2* knockdown in human MDMs blunted immune responses to *Mtb* and BCG [30,53], and knockout of murine *Nod2* decreased immune responses of murine macrophages and DCs to *Mtb* [15,18]. Mouse alveolar macrophages (AMs) required functional NOD2 for the full BCG immune response [22]. Likewise, cytokine production in response to the mycobacterial vaccine *Mycobacterium indicus pranii* (*Mip*) was reduced with *Nod2* knockdown in murine peritoneal macrophages [40].

NOD2 was necessary for complete tumor necrosis factor (TNF) production in response to BCG, *M*. *smegmatis*, *Map*, and 2 other *namH*-possessing actinobacteria (*Nocardia asteroides* and *Rhodococcus equi*), in contrast to bacteria not having *namH* (*Escherichia coli*, *Staphylococcus aureus*, and *Streptomyces* sp.) [25]. In studies of complete Freund’s adjuvant (CFA, an emulsion containing dead mycobacteria), immune responses were dependent on both bacterial *namH* and murine *Nod2* [24,64]. Collectively, these results support that the distinct chemical structure of mycobacterial peptidoglycan (i.e., the *N*-glycolylation of muramic acid by NamH) increases the NOD2-dependent immune response. The increased biological activity of *N*-glycolyl MDP to *N*-acetyl MDP was explained by increased NOD2 protein stability [149].

The *M*. *leprae* genome is degenerate [150], indicative of an obligate pathogen with a restricted lifestyle. *M*. *leprae* has pseudogenized *namH*, resulting in production of *N*-acetylated peptidoglycan only [134]. Nevertheless, *NOD2* dependence was demonstrated in the immune response to *M*. *leprae* [28,32,60] and *M*. *leprae*-specific murodipeptides (containing glycine instead of alanine and with variable amidation of the glutamate residue) [60,134], providing grounds for the genetic associations with leprosy. Known *M*. *leprae*-associated *NOD2* SNPs are noncoding or synonymous [105–109]; if their effect is on the expression level of NOD2, which should be investigated, the system may be more vulnerable to a weaker (*N*-acetyl) MDP ligand. *NOD2* polymorphisms were more strongly associated with multibacillary than paucibacillary infection, and with type II (antibody-mediated) than type I (cell-mediated) lepra reactions [105,106]. *Mtb* is more transmissible in those with cavitary disease requiring cell-mediated immunity [151,152]. This differential flavor of immune response required for efficient transmission may explain the evolutionary decay of *namH* in *M*. *leprae*.

We could find only 2 articles in the literature having data to suggest that *N*-acetylated MDP and PGN are more immuno-stimulatory. One study using PBMCs only compared MDPs and *namH* genotypes at single doses [54]. Another using HEK293T cells [153] is at odds with 3 different HEK studies [25,149,154]. There are at least 9 articles directly supporting greater potency with *N*-glycolylation: *N*-glycolyl MDP has shown greater potency in HEK cell reporter assays [25,149,154], RAW cells [25,27], primary mouse cells [25,64,155], primary human cells [53], and in vivo mouse models [25,156]. Bacteria possessing NamH elicited a greater immune response in mouse cells [25,46], in vivo mouse models [25,64], and human cells [46]. The conflicting results suggest that timing, MDP amount, or cell type may be critical factors in understanding the NOD2 response to mycobacteria, and future studies should test these factors to uncover the mechanisms of greater *N*-glycolyl MDP activity. An interaction between NOD2 and mycobacterial sulpholipid-1 was also reported [42], but this does not diminish the importance of the NOD2–mycobacterial MDP interaction.

## Expression of NOD2 during mycobacterial infection

Monocytes, MDMs, and AMs express NOD2 at the mRNA and protein levels [30,37]. In one of the first studies on *NOD2* expression during *Mtb* infection, humans with active TB compared to uninfected individuals had similar *NOD2* transcript abundance relative to control transcripts in pulmonary leukocytes; 2 outliers with severe TB had exceptionally high *NOD2* expression [19]. *NOD2* expression increased 2.3-fold in PBMCs following antituberculous treatment but did not significantly change when experimentally infecting PBMCs of healthy controls with various mycobacteria [19]. Another study using BCG-infected mice showed that AMs down-regulated NOD2 expression within 2 weeks of infection [20]. BMDMs and J774A.1 cells infected with live *Mtb* up-regulated *Nod2* transcripts up to 10.6-fold within 24 hours of infection [61]. *NOD2* and *RIPK2* mRNA were found to be up-regulated 3- to 4-fold in skin tissue samples from people with leprosy compared to controls [34], and the frequency of NOD2-positive cells was 3 times higher in tuberculoid than in lepromatous lesions [41]. *Nod2* mRNA expression increased following *M*. *leprae* infection of mouse footpads and RAW264.7 cells [51]. Mouse peritoneal macrophages treated with *Mip* up-regulated *Nod2* mRNA expression 3- to 4-fold [40], and goldfish macrophages up-regulated NOD2 mRNA expression 6-fold in response to the fish pathogen *M*. *marinum* [45]. Diverse mycobacterial infections appear to be associated with increased NOD2 levels, presumably increasing bacterial sensing, which requires experimental confirmation.

From the point of view that loss-of-function NOD2 variants are detrimental to the host’s immune response, having an increased abundance of functional NOD2 (and by conjecture increased NOD2 signaling) would be beneficial. NOD2 expression or signaling was shown to be dependent on *NOD2* polymorphism [72], vitamin D levels [49], hypoxia [42], HIV preexposure prophylaxis [71], and (nonsignificantly) type 1 diabetes status [157], potentially explaining how such noninfectious factors have been associated with TB.

## Cell signaling effects of NOD2 during mycobacterial infection

**Fig 3:**
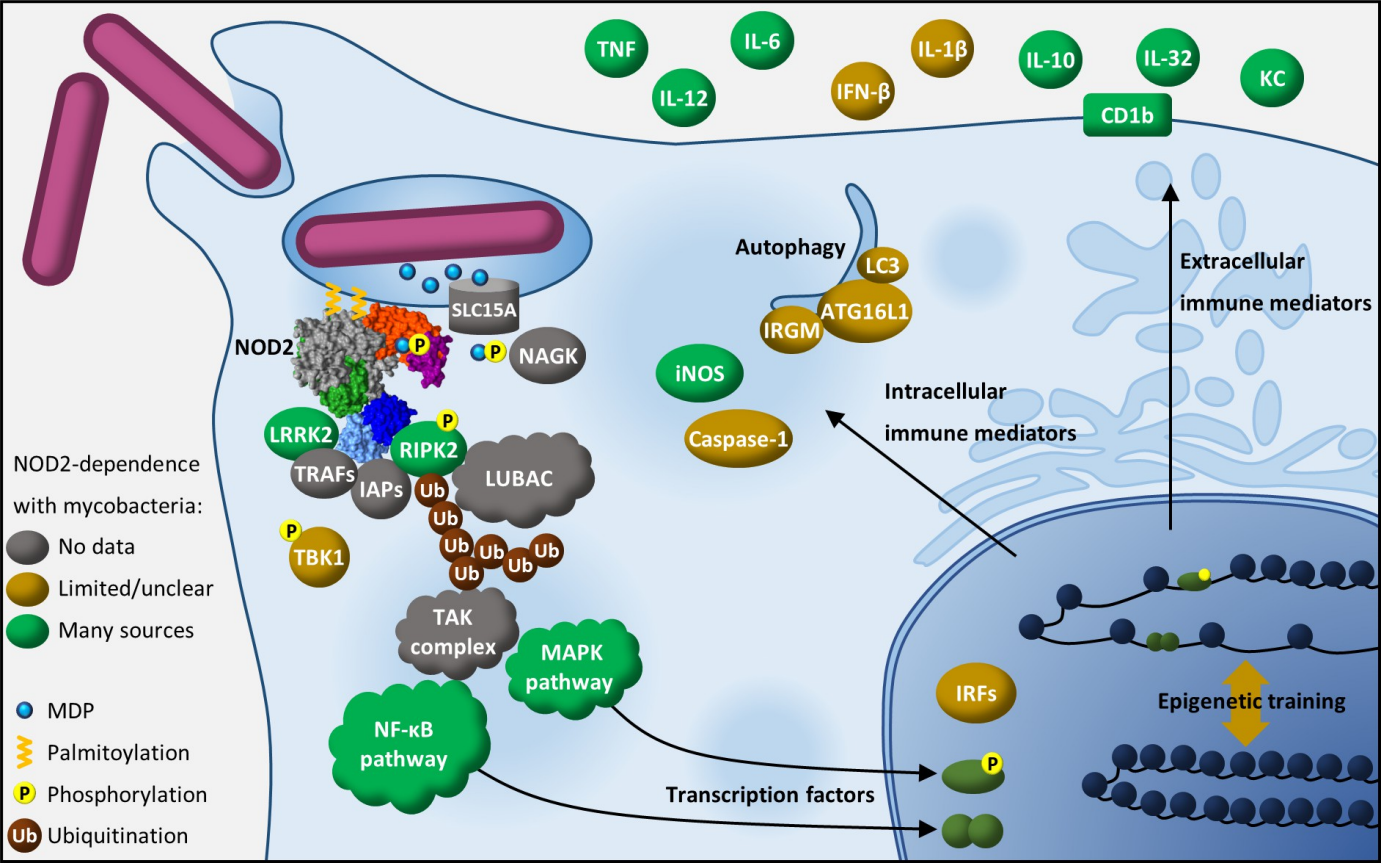
NOD2 signaling during mycobacterial infection. Cartoon depicting mycobacteria (fuchsia bacilli) being engulfed and sensed by a macrophage through NOD2. Muropeptides including the MDP motif exit the phagosome via a SLC15A family transporter. Upon entering the cytosol, MDP is phosphorylated by NAGK and subsequently sensed via NOD2, which has been recruited to the membrane by palmitoylation from ZDHHC5 (not shown). RIPK2 is phosphorylated and ubiquitinated, mediating signaling cascades and culminating in NF-κB and MAPK transcription factors translocating to the nucleus to regulate inflammatory gene transcription. Various immune mediators, both intracellular and extracellular in activity, result from NOD2 signaling. The degree of evidence for involvement of the depicted elements in the NOD2-dependent immune response to mycobacteria is indicated semiquantitatively (no data, limited/unclear, and many sources). ATG16L1, autophagy-related 16 like 1; IAP, inhibitor of apoptosis; iNOS, inducible nitric oxide synthase; IRF, interferon regulatory factor; IRGM, immunity-related GTPase M; KC, KC(CXCL1); LRRK2, leucine-rich repeat kinase 2; LUBAC, linear ubiquitin chain assembly complex; MAPK, mitogen-activated protein kinase; MDP, muramyl dipeptide; NAGK, *N*-acetylglucosamine kinase; NOD2, Nucleotide-binding Oligomerization Domain-containing 2; RIPK2, receptor-interacting protein kinase 2; TBK1, TANK-binding kinase 1; TNF, tumor necrosis factor; TRAF, tumor necrosis factor receptor-associated factor.

From our literature search and knowledge as stated above, in this section, we summarize what has been demonstrated in experiments testing the interaction between mycobacteria and NOD2 regarding intracellular signaling and intercellular (e.g., cytokine) signaling. NOD2 signaling during mycobacterial infection elicits a myriad of immune effectors from the cell, and the results of our literature analysis are summarized in Fig 3. The first studies to demonstrate that mycobacterial sensing was dependent on NOD2 used NF-κB–based reporter systems [15,17,32]. Live *Mtb* caused RIPK2 polyubiquitination in infected murine BMDMs independent of MyD88 and TLR2/4 [158], but dependent on NOD2 [27]. In human MDMs infected with *Mtb*, and PBMCs infected with *Map*, NOD2-dependent phosphorylation of RIPK2 was observed [30,54], which is required for signaling like ubiquitination [159,160]. During *M*. *abscessus* infection of BMDMs, IκBα phosphorylation and degradation were *Nod2* independent, whereas phosphorylation of p38, JNK, and, possibly, ERK was *Nod2* dependent [62]. *N*-glycolyl MDP was more potent than *N*-acetyl MDP in a HEK293 cell NF-κB assay, and in RIPK2 polyubiquitination and phosphorylation of IκBα and JNK but not p38 in RAW264.7 cells [25]. Sensing of mycobacteria and mycobacterial MDP through NOD2 significantly contributes to NF-κB and/or MAPK signaling.

Numerous studies have reported significant NOD2 dependence of TNF production from various types of human and mouse cells in response to different mycobacteria [15,17,18,22,25–27,30,32,46,48,58,59,62,66,74]. TNF is critical for host resistance to mycobacterial infection: Animal studies of *Mtb* or BCG infection using knockouts or anti-TNF demonstrated that this pathway is essential for survival [161–164]. Additionally, humans receiving anti-TNF therapy are at heightened risk of developing TB [165]. Other Th1 cytokines are also important in mycobacterial immunity. IFN-γ is essential for the survival of mice infected with *Mtb* [166,167], restricting the extrapulmonary bacterial burden [168]. IL-12p40 is also critical during murine TB [169]. Moreover, humans with genetic mutations in the IFN-γ/IL-12 pathways are likely to develop disseminated BCG postvaccination, NTM infection, or later *Mtb* infection [170]. Multiple reports support that IL-12 responses require functional NOD2. Knockout of *Nod2* in BMDMs reduced IL-12p40 release by about half during *Mtb* infection [18]. A contemporary study showed the same reduction in IL-12p40 production with *Nod2*-KO naive AMs infected with BCG ex vivo, as well as lung macrophages from BCG-infected mice restimulated ex vivo with BCG [22]. With *M*. *smegmatis* intraperitoneal immunization, *Nod2* and *namH* were necessary for full splenocyte IL-12p40 secretion 14 days later upon rechallenge with the same bacteria (the number of IFN-γ–producing cells and total cytokine release were also *Nod2* and *namH* dependent) [25]. During murine *M*. *avium* infection, *Nod2*-KO resulted in significant reductions in IL-12p40 and IFN-γ transcripts in splenocytes compared to wild-type (WT) controls at 100 days postinfection; IFN-γ in splenocyte supernatant after restimulation with *M*. *avium* was greatly reduced with *Nod2*-KO at the same time point, but not TNF [48]. *M*. *avium*-infected BMDM IL-12p70 production was also dependent on *Nod2* in this study [48]. These results on Th1 cytokines are also reflected with mycobacterial adjuvants: The generation of antigen-specific CD4+ and CD8+ IFN-γ–producing T cells in spleens of mice immunized with CFA required *Nod2* [24]. We demonstrated that both functional *namH* and *Nod2* are required for CFA-induced antigen-specific CD4+ T cells, which secrete IFN-γ and/or IL-17A, and that about half of the adjuvancy of mycobacteria in CFA can be recapitulated with synthetic *N*-glycolyl MDP and a minimal motif of the mycobacterial glycolipid trehalose-6,6′-dimycolate (TDM), which signals through Mincle [64]. Thus, NOD2 contributes to promoting the Th1 response to mycobacteria.

Mycobacteria-induced IL-6 was significantly dependent on NOD2 in human and mouse cells in multiple reports [18,58,59,62,66,74], with corroborating nonsignificant trends in others [15,31]. In but one study investigating the Th1/17-inducing capacity of CFA, injection site IL-6 and IL-23a transcription were independent of *Nod2*, which may be specific to this model or tissue [43]. NOD2-dependent IL-6 could be contributing to the generation of Th17 cells. We have shown that the generation of IL-17A–producing T cells upon CFA immunization depends on *Nod2* [64]. *Mtb* PGN plus TDM, or *N*-glycolyl MDP plus synthetic TDM, are sufficient to generate antigen-specific IL-17A–producing CD4+ T cells in mice [43,64]. Two reports of IL-17A production from human PBMCs infected with *Mtb* were inconsistent on *NOD2* dependence, comparing functional *NOD2* to 3020insC *NOD2* [29,58].

Excess type I IFN drives susceptibility to *Mtb* in mice [171] and is associated with TB disease in humans [172]; it is thought to be detrimental for the *Mtb*-infected host in most (but not all) contexts [173]. *Nod2*-dependent type I IFN production in response to *Mtb* was demonstrated in 2 studies using murine BMDMs. NOD2 and RIPK2 were required for about half of the IFN-α and IFN-β transcription and secretion within 4 hours of infection with *Mtb* [23,27]. Moreover, the *Nod2*-dependent IFN-β transcriptional response appears to be mediated through IRF5, not IRF3, and may require *Mtb* to have cytosolic access via the ESX-1 secretion system, given that a ΔESX1 mutant was incapable of inducing IFN-β transcription [27]. However, a later study challenged this model, showing that IRF3 was absolutely essential for the type I IFN response to *Mtb*, while ESX-1 was still necessary, and that absence of *Nod2* was not associated with a significant decrease of IFN-β transcription in BMDMs [39]. RIPK2 was also dispensable for IFN-β transcription in *Mtb*-infected BMDMs [21]. Apart from *Mtb*, TBK-1 phosphorylation and IFN-β transcription in *M*. *abscessus*-infected BMDMs was *Nod2* dependent, and recombinant IFN-β promoted bacterial clearance in vivo [68]. Clearly, in some situations of mycobacterial infection, which are important to define, type I IFN is NOD2 dependent. Therefore, future studies must determine when (e.g., early/late) and where (e.g., lung/extrapulmonary) NOD2-dependent type I IFN exists, and whether it is beneficial or harmful in that context.

Multiple studies using IL-1 receptor knockout mice infected with *Mtb* have established the importance of IL-1 signaling [174–176]. IL-1R signaling was critical to limit pathological type I IFN in murine TB [171]. IL-1α/β knockout mice were similarly or less susceptible than the IL-1 receptor knockout [164,177,178]. In PBMCs responding to *Map* or *Mtb*, full IL-1β secretion required functional *NOD2* (without 3020insC) [17,26,58]; HEK293T cells infected with *M*. *leprae* required a *NOD2* expression plasmid for *IL1B* transcription [32]. An siRNA-mediated *NOD2* knockdown in MDMs reduced IL-1β secretion by over 80% upon *Mtb* and BCG infection [30]. However, an siRNA-mediated *NOD2* knockdown (only 60% reduction in *NOD2* transcript abundance) in THP-1 cells resulted in insignificant difference in IL-1β production following *Mtb* infection [44]. IL-1β data are less consistent in mouse cells. With murine macrophages (peritoneal and BMDMs), the release of IL-1β upon infection with *Mtb*, BCG [30] and *M*. *abscessus* [62], and *Il1b* transcription with *Mip* [40] was NOD2 dependent. However, NOD2 independence in IL-1β secretion upon *Mtb* infection was shown with BMDCs [35] and BMDMs [36,66], as well as *Il1b* transcription in CFA-immunized skin biopsy [43]. As both transcript and protein data are inconsistent across studies, a difference in caspase activity between cell types cannot fully explain the conflict. Other species/cell differences may explain the results, which require further experimentation. Nevertheless, NOD2 and IL-1β production are linked in certain contexts.

While IL-10 has the capacity to dampen immunopathological responses, its inhibition of protective immune system functions is thought to contribute to mycobacterial susceptibility [179]. NOD2-depdendent IL-10 was reported in human PBMCs treated with sonicated *Mtb* and *Map* [15,17]. A corroborative trend was observed in another study of *Map* infection of human monocytes [31]. Murine BMDCs also required functional *Nod2* for maximum IL-10 production in response to *Mtb* infection [18]. However, AMs infected with BCG ex vivo and lung APCs from BCG-infected mice restimulated ex vivo with BCG or *Mtb* culture filtrate did not depend on *Nod2* for IL-10 production [22]. Splenocytes from mice infected with *M*. *avium* also did not significantly depend on *Nod2* for IL-10 production when reexposed ex vivo to *M*. *avium* [48]. Cell type–specific dependence on NOD2 could explain the inconsistencies.

*Mtb* and BCG can induce IL-32 production from PBMCs (mainly from monocytes) by a Caspase-1/IL-18/IFN-γ–dependent pathway [180]. IL-32 appears to be protective during *Mtb* infection [181,182]. NOD2 contributes to IL-32 production in response to *M*. *leprae*: MDP was able to drive IL-32–dependent differentiation of human DCs, promoting CD1b expression, and defects in this pathway correlated with *M*. *leprae* disease, providing biological grounds for the association of *NOD2* polymorphisms with leprosy [41]. The dependence of the *M*. *leprae* immune response on NOD2 was subsequently demonstrated, where human monocytes’ up-regulation of IL-32 production and CD1b expression to *M*. *leprae* and *M*. *leprae*-derived muropeptides required NOD2 [60].

There are limited data on the NOD2 dependence of other immune effectors elicited by mycobacteria. Production of IL-22 by *Mtb*-infected PBMCs required functional NOD2 [58]. In CFA-immunized mice, antigen-specific splenic IL-4 plus serum IgG1 and IgG2c required *Nod2* [24]. For chemokines, *Nod2* dependence was shown for RANTES from *Mtb*-infected BMDMs, and KC (CXCL1) from *Mtb* lysate-stimulated BMDMs [58] or *M*. *smegmatis*-infected murine peritoneal cavity [25].

## NOD2 has no mycobactericidal effect per se

In this section, we summarize the mechanisms of mycobacterial control/killing, which have been examined as a function of NOD2 status: nitric oxide (NO), bacterial burden during in vitro cellular infections, autophagy, and host cell death modality. Inducible nitric oxide synthase (iNOS), an important source of bactericidal NO, is critical for host resistance to *Mtb* infection [183–185]. *N*-glycolyl MDP was able to induce expression of iNOS and NO production in human MDMs to levels similar to or greater than *N*-acetyl MDP or IFN-γ, and iNOS expression was NOD2 dependent during *Mtb* infection [53]. During *Mtb* infection of IFN-γ–stimulated BMDMs, generation of the NO product nitrite was *Nod2* dependent, but bacterial control was affected only by IFN-γ and not *Nod2* genotype. *Nod2* was also required for nitrite production in response to heat-killed *Mtb* [18]. In a study of BCG-infected mice, lung macrophages examined ex vivo 4 weeks postinfection required *Nod2* for maximum nitrite production [22]. IFN-γ–stimulated BMDMs infected with *M*. *abscessus* similarly required *Nod2* for nitrite production [62].

Multiple studies have tested the NOD2 dependence of in vitro bacterial control. Human MDMs required NOD2 for control of *Mtb* and BCG, while *Nod2* did not help murine BMDMs control *Mtb*, although it was necessary for BCG control [30]. Control of *Map* in monocytes from Crohn’s disease patients heterozygous for the common *NOD2* mutations was not significantly hampered compared to patients homozygous for functional *NOD2*, suggesting *NOD2* haplosufficiency or no role for NOD2 in *Map* control [31]. IFN-γ and iNOS were required for *Nod2*-dependent control of *M*. *abscessus* by BMDMs [62]. Other mouse studies did not support a role for *Nod2* for in vitro bacterial control of *Mtb*, but they used BMDMs that were not stimulated with IFN-γ [18,66], as in Brooks and colleagues [30]. The requirement of IFN-γ for BMDMs to engage NOD2 in mycobacterial control should be further investigated. Macrophage ontogeny may be critical and informative about NOD2-dependent control of mycobacteria, but these elements have not yet been directly studied together.

*Mtb* can also be controlled through the regulation of cell death modality [186] and autophagy [187]. Autophagic control of *Mtb* in human AMs can be enhanced with supplementary MDP, associated with the recruitment of ATG16L1, IRGM, and LC3 to the *Mtb*-containing phagosome [37], and NOD2 is known to contribute to autophagy by RIPK2-dependent and/or independent mechanisms [188,189]. However, data directly demonstrating the importance of physiological NOD2 signaling to autophagic control of mycobacteria is lacking. During infection with avirulent *Mtb* (H37Ra), inclusion of a dominant negative RIPK2 construct in THP-1 cells did not significantly alter apoptosis, despite the fact that MDP was capable of suppressing apoptosis in the same uninfected cells [16]. Another study examining NOD2 during virulent *Mtb* (H37Rv) infection of murine BMDCs found no significant difference in apoptosis between WT and *Nod2*-KO cells [35]. HEK293T cells required overexpression of NOD2 during stimulation with live *M*. *leprae* to optimally up-regulate *CASP1* expression [51]. Recently, an examination of *LRRK2* and *NOD2* polymorphisms in BCG-infected RAW264.7 cells revealed that while NOD2 and LRRK2 jointly contribute to ROS production, only LRRK2 genotype had a significant impact on apoptosis [74]. We could find no evidence directly linking NOD2 per se to apoptosis during mycobacterial infection.

## Murine mycobacterial infection as a function of NOD2

In the first reported *Mtb* infections of *Nod2*-deficient animals (aerosol infections with 35 CFU of strain 1254 or 1,500 CFU of H37Rv), the burden of *Mtb* in the lungs of *Nod2*-KO C57BL/6 mice was indistinguishable from WT mice up to 8 weeks post aerosol infection, with no obvious histopathological differences and only a small increase in serum IL-12p40 in the knockout at earlier time points [18]. In a subsequent study using aerosol infection with 100 CFU of H37Rv, the lack of difference in bacterial burden up to 8 weeks postinfection was confirmed, but *Nod2*-KO mice (C57BL/6) had less pulmonary leukocyte infiltration than WT during *Mtb* and BCG infections [22]. Upon further examination of BCG infection, *Nod2*-KO mice had a pulmonary reduction in CD4+ and CD8+ T cell frequency compared to WT, plus significantly reduced TNF and IFN-γ in BAL fluid reflected by defective cytokine responses in the spleen and lymph nodes at 4 weeks postinfection. Pulmonary *Mtb* burdens at 24 weeks postinfection with 400 CFU of H37Rv were significantly higher in *Nod2*-KO animals compared to WT, and survival was reduced. We recently reported again on *Mtb*-infected *Nod2*-KO mice (C57BL/6) while investigating dual (NOD2 and Mincle) PRR knockout: After aerosol infection with 15 CFU H37Rv, there was no significant effect on bacterial burden and pulmonary leukocyte infiltration by 6 weeks postinfection [73]. After aerosol infection with 31 CFU H37Rv, the survival of *Nod2*-KO and *Nod2*-KO *Mincle*-KO (double knockout) animals was similar (291 and 281 days, respectively) and significantly reduced compared to WT (over 363 days), while *Mincle-KO* survival was intermediate (324 days), suggesting that *Nod2* deficiency was the driver of hastened mortality in the double knockout. Interestingly, increased pulmonary cell death with distinct necrotic foci occurred in the lungs of only some of the *Nod2*-deficient animals upon TB-prescribed euthanasia [73]. These studies confirm that *Nod2* plays an important, albeit delayed, role in host resistance to *Mtb*. The pulmonary necrosis phenotype should be further investigated as mechanism of increased susceptibility to *Mtb* in *Nod2-*deficient animals.

C57BL/6 murine infection with a H37Rv *namH* mutant of *Mtb* showed no differences in pulmonary nor splenic bacterial burden up to 12 weeks post aerosol infection with approximately 30 CFU, and pulmonary inflammation was indistinguishable from WT *Mtb*-infected animals at week 3 [46]. Comparing survival as a function of *namH* in susceptible C57BL/6-DBA2 F1 hybrid mice, no significant differences were obtained. In an experiment with C57BL/6 *Rag1*-KO mice (emphasizing the conditions of early infection where innate immunity predominates), mice aerosol infected with 51 CFU of the *namH* mutant *Mtb* survived significantly longer than those with 34 CFU of WT *Mtb* [46]. In this case, a less inflammatory pathogen permitted slightly prolonged survival in a mouse model where mortality occurs as inflammation compromises lung function.

A recent study described an *N*-deacetylase in *Mtb*, encoded by *Rv1096*, which is expected to remove *N*-acetyl groups from the PGN polymer at GlcNAc and/or MurNAc. When *Rv1096* was ectopically expressed in *M*. *smegmatis*, NOD2- and TLR-dependent reduced inflammatory responses plus prolonged bacterial persistence were observed during cellular and C57BL/6 mouse infections. The corresponding knockout in *Mtb* H37Rv had corroboratory effects, although only data demonstrating overall partial deacetylation of peptidoglycan with the *M*. *smegmatis* mutant were presented using Fourier-transform infrared spectroscopy; no detailed chemical structure was confirmed [66]. This exemplifies the trade-off of reduced NOD2-mediated acute inflammation with increased bacterial persistence.

Like *Mtb* infection, bacterial burden in C56BL/6 mice intravenously infected with 10^6^ CFU of *M*. *avium* was unaffected by *Nod2* genotype at 30 and 100 days postinfection, yet immunological defects were detected along the Th1 axis [48]. Liver granuloma size plus lymphocyte and granulocyte numbers were reduced in *M*. *avium*-infected *Nod2*-KO mice compared to WT at day 100 [48]. Although the organ and phenotype are different, together with the aforementioned pulmonary necrotizing foci, the data point to *Nod2* in the long-term regulation of mycobacterial lesions such as the granuloma. As discussed in our *Mtb* study, pulmonary necrotizing foci are a pathological characteristic of *Mtb* infection in the “Kramnik mouse” [190,191] which has been linked to excessive type I IFN [171]. An NTM model of infection could permit a more rapid and/or convenient investigation of NOD2 in this phenotype.

High-dose (1.5 × 10^7^ CFU) intranasal infection with *M*. *abscessus* proceeds differently than *Mtb* and *M*. *avium* infections: *M*. *abscessus* is cleared from the lungs of immunocompetent mice. However, with *Nod2*-KO, clearance lagged behind WT C57BL/6 mice by about 1 log up to 20 days postinfection [62]. Concomitantly, pulmonary histopathology was worse in *Nod2*-KO. IFN-γ remained reduced in lung homogenates of *Nod2*-KO, while TNF and IL-6 were dysregulated [62]. The results suggested insufficient immunity early resulting in pathological inflammation later, as bacterial clearance was not efficient. In a subsequent *M*. *abscessus* study of the same model, *Nod2*-KO in C57BL/6 mice resulted in defective NO production and bacterial control in the lung, both of which could be rescued with recombinant IFN-β, suggesting a NOD2-IFN-β-NO pathway of *M*. *abscessus* control early in infection [68].

### NOD2 and mycobacteria beyond infection

The potent adjuvant activity of mycobacteria has been renowned since Jules Freund invented his eponymous adjuvant in the mid-20th century. Various mycobacteria were discovered to contain *N*-glycolylated muropeptides from 1969 to 1970 [135,192–195], and by the 1970s, the adjuvant activity of mycobacteria was attributed to MDP [196]. The adjuvant effect of mycobacteria has been recapitulated with purified TDM plus PGN in IFA [43], and more specifically with synthetic TDM (GlcC14C18) plus *N*-glycolyl MDP in IFA to generate experimental autoimmune encephalomyelitis (EAE) [64], demonstrating that *N*-glycolylated PGN/MDP is sufficient to recreate the adjuvant effect in combination with one other mycobacterial MAMP. Synergistic activation of the immune response in vitro and in vivo was also reported between MDP and purified *Mtb* Hsp70, a putative TLR/MyD88 MAMP [52,197]. While *Nod2*-KO and *Ripk2*-KO mice are resistant to EAE induced with CFA [33], one study reported that NOD2 played an immunoregulatory role in CFA-induced experimental autoimmune uveitis through T cell–intrinsic, RIPK2-independent NOD2 activity [67]; this curious result still affirms a role for NOD2 in the mycobacterial adjuvant effect.

Antimycobacterial MDP synergy was explored with the unnatural combination of purified LPS and *N*-glycolyl MDP (gram-negative and actinobacterial MAMPs) to successfully boost various BMDC effector functions during *Mtb* infection [55–57,69]. Similar results were reported with murine mesenchymal stem cells [70]. Prophylactically, a NOD2-RIG-I dual activating small molecule called Inarigivir enhanced BCG vaccine protection during murine *Mtb* infection [65]. Even alone, *N*-glycolyl MDP administered during intranasal infection with *M*. *abscessus* or influenza A virus improved pulmonary clearance and reduced pathology [62,198].

Trained innate immunity elicited by BCG may be key to the utility of the bacillus [38,199]. Cancer is being targeted nonspecifically with BCG [200], *Mip* [201,202], and the *Mtb* extract Z-100 containing PGN [50,203]. With monocytes homozygous for the *NOD2* frameshift mutation, trained innate immunity was defective after in vitro BCG [38] or gamma-irradiated BCG training [47]. Corroborative results were obtained using an RIPK2 inhibitor, and MDP alone induced a similar effect as BCG [38]. Thus, mycobacterial stimulation of NOD2 may contribute to trained innate immunity. However, double knockout of *Nod1* and *Nod2* did not significantly reduce the ability of BCG to control murine *Mtb* infection, indicating that BCG provides *Nod2*-independent protection too [63]. Targeting the NOD2 pathway in the classical vaccine approach, treating infection and trained-innate immunity, continues to be promising and can be informed by studies on mycobacteria but requires further research before clinical applications can be tested.

## Conclusions

Nearly 2 decades of research have asserted a significant relationship between mycobacteria and NOD2. Polymorphisms in NOD2 have been associated with mycobacterial diseases. The mycobacterial NOD2 ligand *N*-glycolyl MDP is unique, shows enhanced NOD2 activity, and contributes significantly to the immune response. NOD2 sensing of mycobacteria manifests as heightened production of various immune effectors and likely contributes to bacteriologic control. NOD2 is protective against death during *Mtb* infection of animals by an unknown mechanism. Future studies will need to focus on NOD2-dependent mechanisms of immunopathogenesis (potentially type I IFN and/or cell death) to uncover how absence of functional NOD2 leads to altered disease phenotypes. Conversely, promoting the NOD2 pathway has demonstrated immunotherapeutic potential and merits further development.
